# Ecosystem services provided by a complex coastal region: challenges of classification and mapping

**DOI:** 10.1038/srep22782

**Published:** 2016-03-11

**Authors:** Lisa P. Sousa, Ana I. Sousa, Fátima L. Alves, Ana I. Lillebø

**Affiliations:** 1 Department of Environment and Planning & CESAM - Centre for Environmental and Marine Studies, University of Aveiro, Campus Universitário de Santiago, 3810-193 Aveiro, Portugal; 2 Department of Biology & CESAM - Centre for Environmental and Marine Studies, University of Aveiro, Campus Universitário de Santiago, 3810-193 Aveiro, Portugal

## Abstract

A variety of ecosystem services classification systems and mapping approaches are available in the scientific and technical literature, which needs to be selected and adapted when applied to complex territories (e.g. in the interface between water and land, estuary and sea). This paper provides a framework for addressing ecosystem services in complex coastal regions. The roadmap comprises the definition of the exact geographic boundaries of the study area; the use of CICES (Common International Classification of Ecosystem Services) for ecosystem services identification and classification; and the definition of qualitative indicators that will serve as basis to map the ecosystem services. Due to its complexity, the Ria de Aveiro coastal region was selected as case study, presenting an opportunity to explore the application of such approaches at a regional scale. The main challenges of implementing the proposed roadmap, together with its advantages are discussed in this research. The results highlight the importance of considering both the connectivity of natural systems and the complexity of the governance framework; the flexibility and robustness, but also the challenges when applying CICES at regional scale; and the challenges regarding ecosystem services mapping.

The mapping and assessment of ecosystem services (ES) is one of the core actions (Action 5) of the European Union’s (EU) Biodiversity Strategy, which aims at *“halting the loss of biodiversity and the degradation of ecosystem services in the EU by 2020, and restoring them in so far as feasible, while stepping up the EU contribution to averting global biodiversity loss”*^1^. To support the implementation of Action 5 a working group on Mapping and Assessment of Ecosystems and their Services (MAES) was established^2^,^3^. These efforts place the European Union on the course to achieve its global commitments under the Convention on Biological Diversity, particularly the Aichi Target 11 on conservation of biodiversity and ES of terrestrial, inland water, coastal and marine areas through the expansion of protected areas; and the Target 14 on restoring and safeguarding essential ES.

Approaches to ES mapping are abundant and vary on aim and rational, type of ES analysed, spatial scale, and source of information^2^,^4^,^5^. Several mapping methodologies’ reviews are available in literature^4^,^5^,^6^,^7^,^8^,^9^. According to Martínez-Harms and Balvanera^5^ review, which focus on social-ecological assessments of ES, the most commonly mapped ES are carbon storage, carbon sequestration, food production and recreation. The most frequently used method is the causal relationships based on the understanding of ES and readily available information. Other methods for mapping ES are extrapolation of primary data (e.g., field data, surveys, and census data), expert knowledge, regression models and look-up tables. Regional (10^3^–10^5^ km^2^) and national (10^5^–10^6^ km^2^) spatial scales are the most common analysed; and land cover variables, topographical information and spectral vegetation indices are frequently used as source of information^5^.

The process of mapping and assessing ES requires a first stage of identification and classification of ES, which according to Haines‐Young and Potschin^10^ is foreseen as conceptually and technically challenging. The lack of understanding on a common definition of the ES concept, together with the variety of purposes, applications (e.g. environmental accounting, ES mapping, ES valuing) and disciplines involved (e.g. ecology, sociology, economy, geography), contribute to the debate around ES definition and classification. In this context, in 2009, the European Environment Agency (EEA) promoted the development of a consistent classification of ES – the Common International Classification of Ecosystem Services (CICES) – compatible with the design of Integrated Environmental and Economic Accounting methods. Coordinated by the University of Nottingham, CICES provides a “common base” for comparison across Europe^11^.

This classification system has been adopted by the MAES working group and will be used through Europe, by Member States, in the mapping and assessment of the state of ecosystems and their services^3^.

When applied to complex coastal regions, these mapping and classification approaches must be adapted to their biophysical and sociocultural characteristics, governance framework and to the scale of analysis.

Ria de Aveiro coastal region is a complex socio-ecological system given the multiplicity of institutions, organizations and stakeholders involved in its governance, together with the variety of ecosystems included (marine, freshwater, forest, agro-ecosystems). Therefore, and due to its complexity, at a regional scale, the case study of Ria de Aveiro coastal region presents an opportunity to explore the application of such approaches. In addition, this case study is relevant in the European and national context, not only for the coastal lagoon itself and its rich and diverse natural capital, but also for its central national geographic location in the Atlantic coastal zone, between two metropolitan areas (Porto and Lisbon). Moreover, the presence of the Aveiro harbour (considered as one of the most important maritime gates of the Iberian Peninsula and the south of Europe), which allows and strengthens both the maritime and terrestrial transport connections with a number of national and international cities^12^, turns this coastal area pertinent.

Having Ria de Aveiro coastal region as a show case, the main objectives of this paper are: i) to define the exact geographic boundaries of a complex coastal region; ii) to identify and classify the ES provided; and iii) to map the ES provided by a complex coastal region. In this context, the criteria used to define the case study boundaries, as it involves different ecosystem typologies and a complex governance framework are discussed; the provided ES are identified; and the most suitable indicators that will serve as basis to map the ES provided are defined (Fig. 1). This approach follows the principles of integrated and ecosystem-based management approaches, acknowledging the complexity and the interspecies relationship within ecological systems, but also accounting for social and governance objectives^13^. It aims to contribute to the discussion on ES classification and mapping at regional scale.

## Methods and Materials

### Definition of the geographic area

Geographic boundaries are frequently defined by administrative boarders, (e.g. county, municipality, parish), statistical units (e.g. NUTS - Nomenclature of Territorial Units for Statistics) or by jurisdictional boundaries of planning and management tools through plans and programmes (e.g. strategic planning, land/maritime development, environmental planning). Nevertheless, these boundaries do not always correspond to the geography of human uses, ecosystem processes or boundaries, such as those based on biogeography, oceanography and/or bathymetry^14^. In addition, in the context of an integrated and ecosystem-based management approaches, such boundaries should be taken into account, and a balance between ecological, social and jurisdictional factors should be achieved^15^,^16^.

Therefore, the criteria for defining the study area include:

- Analysis of the territory elements (e.g. land cover, topography, bathymetry) and identification of the physical boundaries of the main studied ecosystems and its interfaces, in order to include significant connective structures of the landscape (i.e., physical relationships that facilitate the link between different elements in the landscape or spatial settings across multiple scales, such as blue and green infrastructures) and thus adopt a system-wide approach^17^;
- Spatial planning and management tools that focus on the study area, particularly at regional or municipal level for land/maritime development, spatial planning, and/or environmental planning;
- Designated areas for nature conservation under international, supranational or national protected areas network (e.g. Ramsar Convention, Natura 2000 network, Nature Parks);
- Administrative and statistical boundaries (e.g. parish, municipality, NUTS III), depending on the detail of the assessment as well as the available information;
- Existence, availability and scale of spatial data, which may condition the enforcement of previous criteria.

### Identification and classification of ecosystem services

Both the ES concept and the ES classification have evolved over time with varying attention for the ecosystem basis or the economic use, as summarized in Table 1.

Despite the extensive debate about its adequacy^18^,^19^,^20^, the Millennium Ecosystem Assessment (MA) definition (Table 1) and classification of ES have been widely adopted. Nevertheless, this definition has also been considered broad and ambiguous^21^,^22^, and alternative definitions have been proposed (Table 1).

In 2009, the EEA fostered and supported the development of CICES, which was adopted by the MAES working group, and will be used by Member States during the implementation of the EU Biodiversity Strategy^3^. For this reason, and given that CICES is consistent with existing and accepted typologies of ES (such as MA^23^ and TEEB^24^), and establishes a basis for comparison between ES assessments in different ecosystems and countries^25^, the final version of CICES (V4.3) is the classification system used in this research.

CICES follows a hierarchical structure as a way to allow its users to select the most appropriate level of detail required to their application. At the highest hierarchical level (called ‘Sections’) there are three broad categories^11^. Below these major ‘Sections’ are nested a series of ‘Divisions’, ‘Groups’ and ‘Classes’, as shown in Table 2. CICES considers the outputs of ecosystems dependent on living processes. Abiotic outputs are classified separately and are hierarchically divided in ‘Sections’, Divisions’ and ‘Groups’. CICES V4.3 does not include abiotic materials and renewable abiotic energy in its main working matrix, but as an accompanying matrix. In the present study both matrixes will be considered.

### Mapping of ecosystem services

From the variety of ES mapping methodologies consulted, the methodology adopted relates to Burkhard *et al.*^4^ and Medcalf *et al.*^26^ methodologies.

Similarly to Burkhard *et al.*^4^, the adopted mapping approach relies on the principle that ecosystems differ in their capacity to provide ES. Besides, as Medcalf *et al.*^26^, it assumes that there is a significant set of available information that can be used to either map a given ES or provide a proxy for the service so that it can be mapped. Like Burkhard *et al.*^4^, this approach uses existing spatial data on habitats and land use/land cover (LU/LC) to demonstrate ecosystems’ capacity to provide ES in a spatial manner. This is complemented with alternative data on management plans, administrative procedures and legal instruments (e.g., designated areas for, or areas restricted to, certain activities), biophysical aspects (e.g., soil typology, evapotranspiration rates, position in the landscape, like next to a watercourse), and human activities (e.g., recreational areas, shellfish collecting areas), similarly to Medcalf *et al.*^26^, enabling to reduce the uncertainty associated and provide maps of actual ES. This approach uses a set of qualitative indicators that are assigned to each ES and abiotic outputs to indicate its presence. A Geographic Information System (GIS) based approach is used to map these ecological, biophysical and socioeconomic features that illustrate provisioning, regulating and maintenance, and cultural services. These maps can be organized by ‘Division’ or ‘Group’ (under CICES classification) depending on their complexity in order to obtain clear and visually attractive maps, easily understandable by technicians, planners and other stakeholder groups.

### Data acquisition

An important phase in ES mapping is data acquisition, database development, and quality assessment^26^,^27^. In order to implement the proposed methodology, several data needs to be collected and analysed regarding physical, ecological, socioeconomic, territorial and structural aspects of the study area and its surroundings. Because ecosystems are one of the primary landscape units that provide ES, it is important to analyse their spatial distribution^28^. Other features that contribute to the study area characterization and the accuracy of ES maps are: morphology of the territory (elevation and bathymetry); river network and water bodies; geology; soil typology; LU/LC; and social and economic data (e.g., population density, buildings and infrastructures), which provide information regarding the major demands alongside the main drivers of change and pressures^29^. Therefore, the resulting geodatabase is composed by multi-source geospatial data in GIS format, which needs to be assessed regarding its spatial coverage, data projections, suitability, date and frequency of updates^27^.

### Study area

Coastal territories are considerably diverse and complex, particularly those integrating estuaries or coastal lagoons such as Ria de Aveiro, as they are transition areas between freshwater and marine systems, and between aquatic and terrestrial systems^30^. Moreover, coastal areas are characterized by an intense human presence and activity, being subject to powerful and growing pressures and impacts.

**Figure 2:**
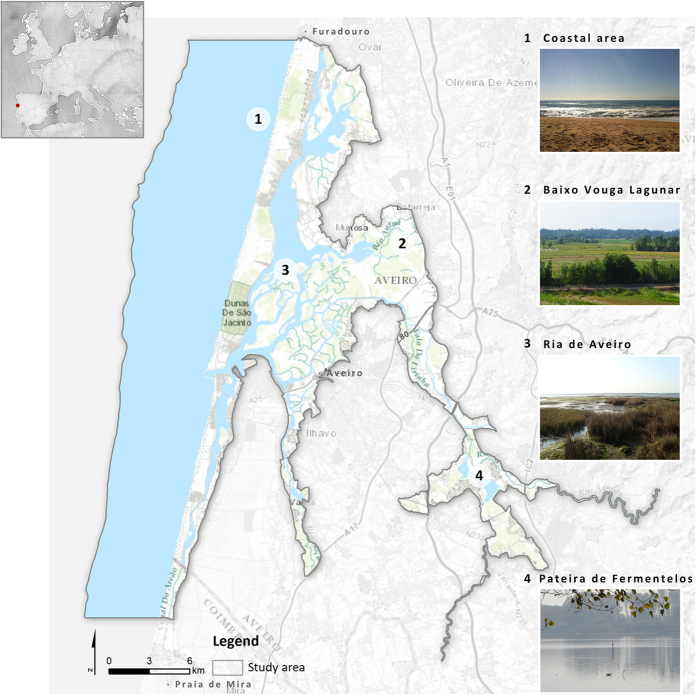
Ria de Aveiro study area and its main territorial elements. Map tiles by Stamen Design (http://stamen.com) under CC BY 3.0 (http://creativecommons.org/licenses/by/3.0/). Data by OpenStreetMap (http://openstreetmap.org), licensed under CC BY-SA. The license terms can be found on the following link: http://creativecommons.org/licenses/by-sa/3.0/. Sources of ESRI World Topographic Map: Esri, HERE, DeLorme, Intermap, increment P Corp., GEBCO, USGS, FAO, NPS, NRCAN, GeoBase, IGN, Kadaster NL, Ordnance Survey, Esri Japan, METI, Esri China (Hong Kong), swisstopo, MapmyIndia, {copyright, serif} OpenStreetMap contributors, GIS User Community. Pictures copyright: 1 ©Lisa Sousa, 2 ©Nuno Rodrigues, 3 ©Ana Lillebø, 4 ©Célia Laranjeira.

The study area is located in the northwest coast of Portugal (40°38′N, 08°45′W) and is integrated in the Vouga river catchment area (368,521 ha) (Fig. 2). Ria de Aveiro coastal region integrates urban areas and a wide range of natural and semi-natural habitats, namely coastal and halophytic habitats (e.g., coastal dunes, coastal lagoon, salt marshes, salt pans, intertidal flats), agro-ecosystems, woodlands and freshwater rivers and lakes^31^. The marine part of the study area is mostly composed by infralittoral fine sand and circalittoral fine sand^32^. Due to the great diversity in habitats and bird species, the study area has been integrated in the Nature 2000 network as a Special Protection Area (SPA) and a Site of Community Importance (SCI). It also incorporates the São Jacinto Dunes Nature Reserve, the Ramsar Site Pateira de Fermentelos Lake, and Águeda and Cértima Valleys. This region generates significant economic benefits originated from land and maritime activities such as agriculture and livestock, fishing, maritime port activity, industry, tourism and recreation^30^.

**Figure 3:**
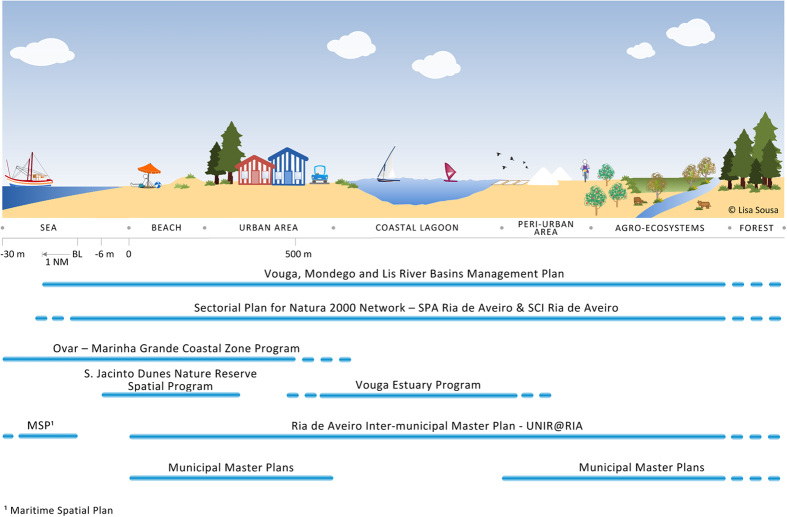
Territorial incidence of case study’s relevant planning instruments for both terrestrial and marine space (after Sousa *et al.*^30^). Drawings were generated with Microsoft Visio Professional 2016.

The governance framework of Ria de Aveiro coastal region is characterized by the involvement of a variety of government departments (e.g., Portuguese Environmental Agency – APA I.P; Regional Development Coordination Commission of the Centre – CCDRC; Aveiro Region Intermunicipal Community – CIRA), non-governmental agencies and other stakeholders (e.g. land-owners, fishermen associations, sports associations). In addition, 11 municipalities have jurisdiction over different parts of the case study^30^,^33^. The spatial planning and management of Ria de Aveiro coastal region is performed by programs and plans of national, regional, inter-municipal and municipal levels. Figure 3 presents a schematic representation of the spatial incidence (marine and terrestrial) and governance framework of the relevant plans and programmes for the study area.

## Results

### The case study boundaries definition

Ecosystem-based management is an integrated approach to management that recognises that human uses and ecosystem health are interdependent. Although it considers ecological, social and cultural objectives, ecological sustainability is the primary goal of management^34^,^35^. To act in accordance with the objectives of this approach, the delineation of the case study boundaries took into account the concepts of structural connectivity (based entirely on landscape structure^36^, i.e. not considering, for now, the functional responses) and complementarity between the different natural and semi-natural systems – marine, transitional, riverine, and terrestrial, including human-shaped ecosystems (agro-ecosystems). However, authors acknowledge the importance of functional connectivity, which is of paramount importance when considering habitats sensitivity and vulnerability, on risk assessment. To comply with the considered concepts, the study area includes, besides the Ria de Aveiro coastal lagoon (the focus of this research): i) the ecological structures considered complementary to the lagoon – e.g., the coastal strip between Furadouro (in the North) and Praia de Mira (in the South) and the lagoon’s margins, responsible for its shape; and the Pateira de Fermentelos freshwater lake, an important wetland from the conservation point of view; and ii) the connective structures – e.g., rivers that flow into the lagoon, riparian corridors, and agricultural fields (such as ‘bocage’) between the lagoon and the Pateira de Fermentelos, which are components of the landscape that can facilitate the biological flows, i.e., which provide important functional connectivity.

Nevertheless, adjustments to the marine and terrestrial boundaries were made in order to match the existing management and spatial planning framework^30^,^37^ (Fig. 3), namely the Vouga Estuary Program (under development) and the Coastal Zone Program (CZP) for the stretch Ovar – Marinha Grande:

- inclusion of the estuary margins (50 m measured from the limit of the water body);
- adjustments in the marine boundary up to a depth of 30 m in order to include the marine area covered by the CZP.

Additionally, adjustments were made to include the limits of the SPA Ria de Aveiro, the SCI Ria de Aveiro, the Ramsar Site Pateira de Fermentelos Lake, and Águeda and Cértima Valleys.

As result of the adjustments, the study area comprises a total area of 62,535ha of which 30,779ha are marine and 31,756ha are terrestrial. Its elevation ranges between 0–20 m in most of the study area, reaching 80 m in the proximity of Pateira de Fermentelos.

### Ria de Aveiro coastal region - ecosystem services identification

A total of 59 ES and abiotic outputs were initially considered in this analysis, following the CICES system for both ES and abiotic outputs. The identification of the ES provided by the study area was based on Barbier *et al.*^38^, Maltby *et al.*^39^, Salomidi *et al.*^40^, ADAPT-MED^41^, Liquete *et al.*^8^, Sousa *et al.*^42^, and Lillebø *et al.*^31^.

For the purpose of presenting the ES identified both in a concise way and without losing detailed information, the study area was divided in four major ecosystem typologies, similar to those defined by Maes *et al.*^3^: coastal waters, transitional waters, freshwaters, terrestrial ecosystems (including agro-ecosystems). ES related to green infrastructures within urban areas were not considered in this study since the current spatial scale does not allow the degree of detail required for that type of analysis.

The ES and abiotic outputs delivered by the Ria de Aveiro coastal region were identified and briefly described in Table 2 (a detailed description is given in Supplementary Tables S1 and S2). Regarding abiotic outputs, it was identified the provision of mineral nutritional substances (e.g. marine salt), associated to transitional waters; non-metallic materials (e.g. sand and gravel), associated to coastal waters; and weather regulation, both associated to transitional and freshwaters.

### Spatial distribution of ecosystem services

A set of qualitative indicators, summarized in Table 3, were identified and assigned to each ES and abiotic outputs. This selection took into account the existence and accessibility of spatial data, which serves as a proxy of the service so it could be mapped. Only the ES and abiotic outputs identified in Table 2 were considered in Table 3; however, not all have an assigned indicator due to the lack of spatial data (e.g. hunting, under the ES class *‘wild animals and their outputs’*) or due to the nature of the ES (e.g. inspiration and sense of place under *‘aesthetic’* ES class, and traditional boats under *‘heritage, cultural’*). Additionally, during the ES and abiotic outputs mapping exercise, some issues were identified regarding the application of the CICES (see Discussion section), resulting in small adaptations of CICES V4.3 table, highlighted in Table 2 with an asterisk. The full list of indicators used to map the ES and abiotic outputs delivered by Ria de Aveiro coastal region, along with the typology and source of data used are given in Supplementary Tables S1 and S2.

**Figure 4:**
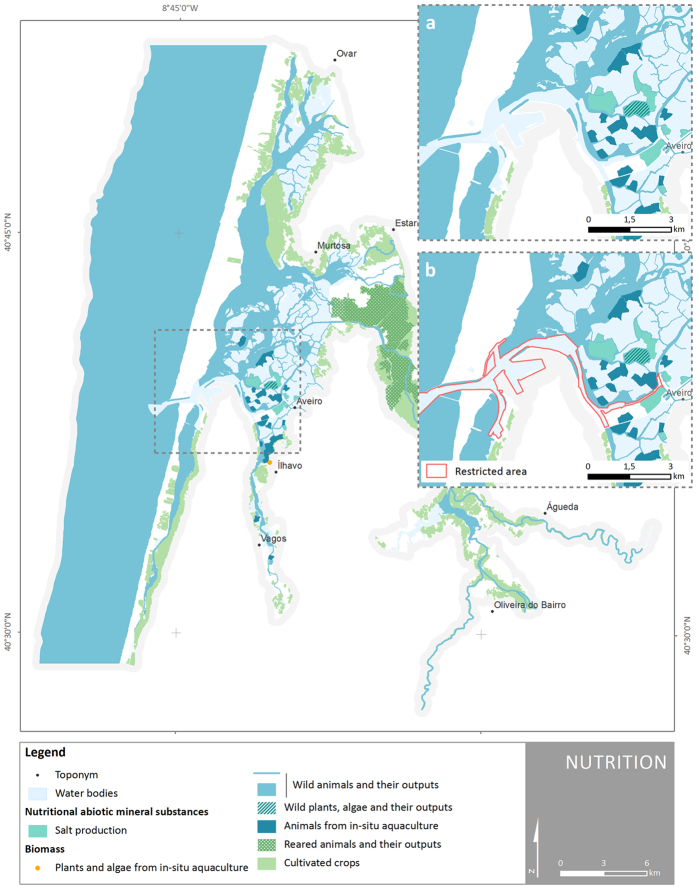
Spatial distribution of ES classes and abiotic outputs present in Ria de Aveiro coastal region under the division ‘nutrition’. (**a**) Detail of Ria de Aveiro central area. (**b**) Fishing restricted area (Public Notice no. 01/2012). Map generated with ArcGIS 10.

Whenever possible and appropriate, data on administrative processes and legal instruments were used in combination with spatial data as a mean to achieve more accurate maps and consistent with the case study reality. This was especially the case of provisioning and cultural services (e.g. *Salicornia* sp. harvesting, fishing and shellfish collecting, archaeological sites, protected areas). The class *‘wild plants, algae and their outputs’* within division *‘nutrition’*, for instance, refers to wild glasswort *Salicornia* sp., which is present in almost all salt pans; however, only one of them is certified and authorized to commercialize it. Thus, only this salt pan was mapped (Fig. 4). Another example is the class *‘wild animals and their outputs’* under the same division. The entire coastal lagoon has potential for fishing; however, this activity is forbidden in certain areas within Aveiro harbour jurisdiction to ensure the safety of navigation, people and goods (Legal Notice no. 01/2012). This results in the exclusion of these areas from the map (Fig. 4).

**Figure 5:**
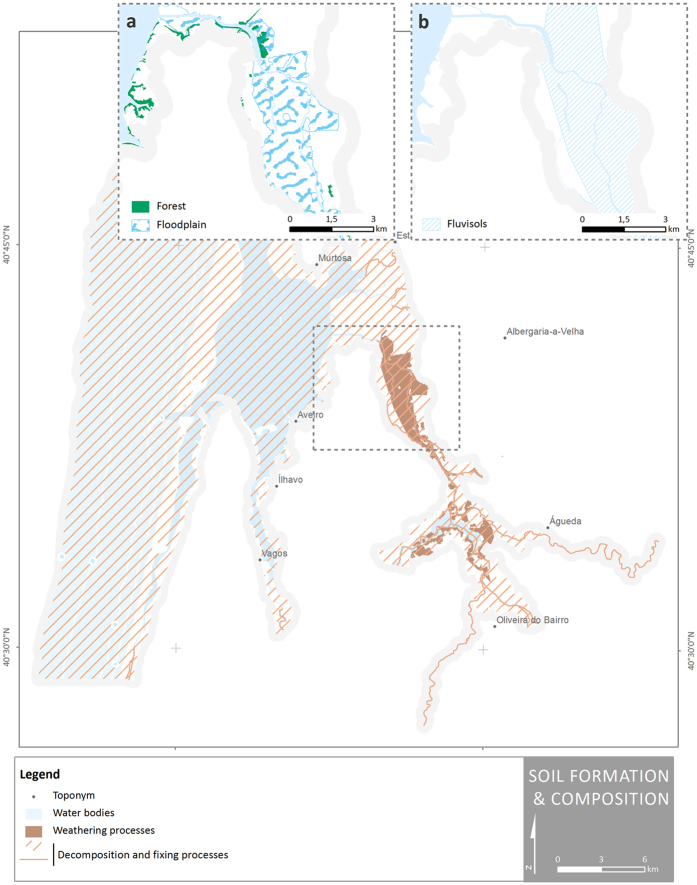
Spatial distribution of ES classes present in Ria de Aveiro coastal region under the group ‘soil formation and composition’. (**a**) Detail of the spatial distribution of forest and floodplain areas. (**b**) Detail of the spatial distribution of fluvisols. Map generated in ArcGIS 10.

The proposed indicators for mapping regulating and maintenance services are dominantly based on the presence and distribution of habitats units; however, in the case of the ES classes *‘hydrological cycle and water flow maintenance’* (within the division *‘mediation of flows’*) and *‘weathering processes’* (within the division *‘maintenance of physical, chemical, biological conditions’*) complementary data was considered to achieve a more accurate representation of the ES. Therefore, for the *‘hydrological cycle and water flow maintenance’* case, areas with higher evapotranspiration rates were considered beyond the riparian and alluvial habitats. For the *‘weathering processes’* case, the type of soil was combined with the land cover and floodplain areas (Fig. 5), i.e. the ES is represented by the areas which integrates both fluvisols and forests, or both fluvisols and floodplain areas. Although the climate and topography have influence in the weathering processes, they were not considered since these factors are quite regular within the study area.

**Figure 6:**
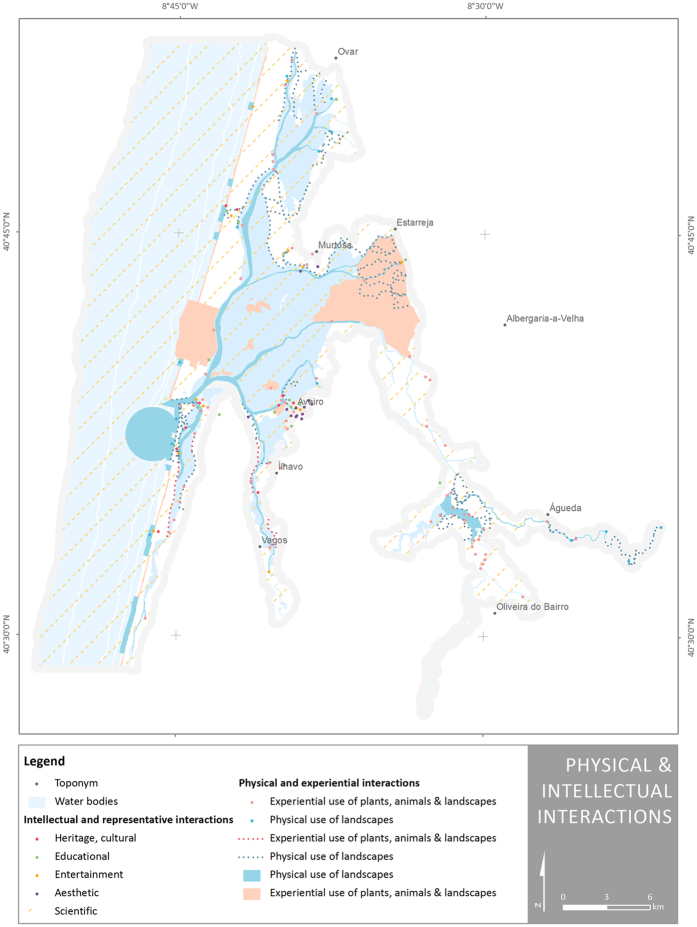
Spatial distribution of ES classes present in Ria de Aveiro coastal region under the division ‘physical and intellectual interactions with biota, ecosystems, and landscapes’. Map generated with ArcGIS 10.

A significant number of cultural services are geometrically represented by points, not only because of the reduced area (even at this scale) occupied by the ES (e.g. subaquatic archaeological sites), but also due to the nature of the ES. The ES class *‘entertainment’*, for instance, refers to ex-situ experiences of the Ria de Aveiro coastal region through festivals and fairs. The location of such festivals and fairs is mapped despite the fact that the service is provided by the existence of salt pans in the coastal lagoon, for example, or the existence of a long tradition of fishing and shellfish collecting in the lagoon. Another example of this technical detail is the use of points to map birdwatching and landscape enjoyment under the *‘experiential use of plants, animals and land-/seascapes in different environmental settings’* class. The locations that allow a better enjoyment of the ES are mapped, despite the fact that is the landscape characteristics, or the birds’ diversity that provide the service (Fig. 6).

## Discussion

The process of defining the study area boundaries, identifying and mapping the ES raised some challenges. The fact that coastal lagoons are interface ecosystems hampers the definition of strict boundaries, essential for the scope of this research. Therefore, there was the need to ensure the link between marine, transitional and riverine systems without losing focus on the main feature of this study, the Ria de Aveiro coastal lagoon. Boundaries definition is a crucial step for the identification and classification of ES, since it enables the understanding on the type of ecosystems present. This is particularly important when considering the existence of land-based recreation activities, for instance, driven by the coastal lagoon (e.g. bird watching, angling, walking), or for example the role of coastal dunes in contributing to the lagoon’s integrity. Moreover, the compliance of the study area with the legal framework is considered an advantage since it enables the integration of these results in the spatial planning and management tools, and facilitates funding acquisition for its implementation or development.

CICES reveals to be suitable to classify ES of complex coastal regions, with minor adjustments according to the scale of analysis, data availability, and its biophysical and sociocultural characteristics. While establishing a common typology of ES – underpinned on clear concepts and principles – CICES is flexible enough to be adapted to different realities, conditions, scales (e.g. Belgium^25^, Ria de Aveiro - this study), and purposes (e.g. mapping, economic valuation). This is possible because i) CICES is based on clear and well defined concepts; ii) it follows a hierarchical structure that allows the adoption of different levels of detail according to the user’s interest; iii) each level of the hierarchical structure has been designed in a way that there is no overlapping nor redundancy; and iv) it is focused on “final” services or outputs from ecosystems that people use or value, in order to avoid double counting^11^,^25^. However, the *‘regulation and maintenance’* section is relatively specific and requires scientific in-depth knowledge about biological and physic-chemical processes, which can be a constraint to its use by decision-makers, technicians, planners, or even by general public.

During the mapping exercise, some issues arose driven by different reasons, which led to minor adaptations to the CICES V4.3 in order to better integrate the case study conditions, scale of analysis and available data:

- Lack of spatially detailed information to distinguish the biota (micro-organisms, algae, plants, and animals) from the ecosystem. This is the case of the ES classes *‘filtration/ sequestration/ storage/ accumulation by microorganisms, algae, plants and animals’* and *‘filtration/ sequestration/ storage/ accumulation by ecosystems’* within the division *‘mediation of waste, toxics and other nuisances’.* Thus, the ES was spatially represented by the ecosystem components (salt marshes and reed marshes, intertidal flats, coastal waters, riparian and alluvial forests) and referred as *‘filtration/ sequestration/ storage/ accumulation by biota and ecosystems’*.
- Lack of supporting information for the ES group *‘water conditions’* regarding the underlying service to achieve such state. In the mapping exercise, this group was excluded since it was considered that the proposed indicators^3^ reflect an ecosystem status based on chemical indicators, specifically the indicators for the trophic status, and not the provided service. Therefore, it reflects the status due to environmental pressures (eutrophication) instead of the provided service. On the other hand, *‘water conditions’* in the scope of the WFD includes also the biological indicators and the priority substances.
- Insufficient knowledge (particularly about the species and their distribution) regarding “natural” biological control has led to the use of the abundance and distribution of alien species or host-species as proxy indicators for the *‘pest control’* (e.g. Maes *et al.*^3^). Again, the proposed indicator reflects the resistance to the environmental pressure (alien species) and not the provided service. However, despite of acknowledging the existence of alien species in the case study (Table 2), this service was not mapped to avoid its misinterpretation or undermine the communication with the technicians, the general public and other stakeholder groups. Nevertheless, authors acknowledge that the spatial distribution of the alien species, and its monitoring, is of paramount importance for the management of ecosystems, and should be considered during a vulnerability assessment.
- Ambiguity and subjectivity associated to the group ‘other cultural outputs’ of the division ‘spiritual, symbolic and other interaction with biota, ecosystems, and land-/seascapes [environmental settings]’. In line with the CICES adaptation to Belgium^25^, this ES group was perceived as part of a valuation analysis and therefore it was excluded from the mapping.

The varying quality, scale and accuracy of the collected data created a barrier, requiring data refinement, reclassification, and projection. Data preparation involved projection to the same coordinate system (in this case ETRS 89 - European Terrestrial Reference System 1989), and data refinement, particularly regarding data on habitat distribution, LU/LC, and seabed benthic habitats, which were collected for the study area from regional, national, and European sources, covering the years 2011, 2007, and 2014, respectively. These pre-existing, but scattered, data was used as starting point to display ecosystems’ spatial distribution, since they are the primary landscape unit that provide ES. Whenever possible, preference was given to more detailed information.

For the terrestrial area overlapping the SPA Ria de Aveiro it was used the habitat map produced by AMBIECO^43^ in the scope of the Characterization Study on Ria de Aveiro Ecological Quality for the Polis Litoral Ria de Aveiro. The habitat map production was based on rectified orthophoto images (from 2005 with spatial resolution of 0.5–1 m) from the Portuguese Geographic Institute (IGP), and complemented with latest information from Bing (2009) and GoogleEarth (2009, 2010, 2011). For the remaining terrestrial area it was used the second level (15 classes) of LU/LC for Continental Portugal (COS2007), produced by IGP^44^. COS2007 was produced based on visual interpretation of rectified and high resolution (50 cm) orthophoto images. COS2007 is in vector format and has a minimum mapping unit of 1ha.

For the marine part of the study area (up to 30 m depth) it was used the benthic habitat map from MESHAtlantic project (last updated in February 2014), available in the European Marine Observation and Data Network (EMODnet) website (http://www.emodnet-hydrography.eu/). The MESHAtlantic project covers over 356,000 km^2^ of seabed habitats of the European North Atlantic Ocean and used a broad-scale mapping method, proposed within the INTERREG MESH project, which is based on available information or on data derived from mathematical models of the marine environment. The broad-scale map is a map of the physical characteristics of the habitats with a 250 m grid resolution (which is roughly equivalent to a scale of 1:1,000,000) and uses the level 4 of the European Nature Information System (EUNIS) classification habitat types^45^.

**Figure 7:**
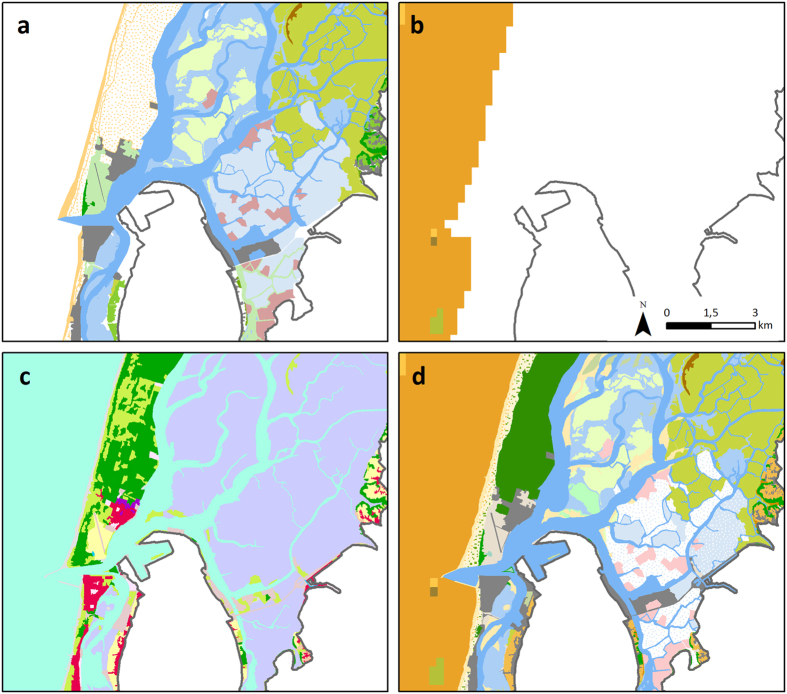
Detail of sources/typologies of data-(**a**) Habitats from AMBIECO/ PLRA, 2011; (**b**) Benthic habitats from MESHAtlantic, 2014; (**c**) COS2007, from IGP, 2010- used to obtain the final habitat map (**d**) for the Ria de Aveiro coastal region. Map generated with ArcGIS 10.

In order to combine these three sources of information, the habitat classification was refined to harmonize the LU/LC taxonomies with the habitat classification (Fig. 7 and Supplementary Table S3).

All these challenges, along with the significant complexity of social-ecological systems and the scientific attempts to cope with that complexity^46^,^47^,^48^, contribute to a certain degree of uncertainty that must be internalized when using and communicating the results. Bellow we identify some of the sources of uncertainty:

- Generalization and categorization used to reduce complex landscapes into a limited number of LU/LC, or habitat classes^47^. In the case study of Ria de Aveiro coastal region, whenever possible, it was given preference to habitat data, which was the most accurate available information. In the absence of more detailed information, the second level of COS2007 and the MESHAtlantic benthic habitat were used.
- Another source of uncertainty is the spatial and temporal mismatches of different sources of data. For example, in Ria de Aveiro coastal region, spatial data from different years and with different spatial resolutions had to be combined in order to fill the spatial gaps in habitat map. Moreover, even the most recent available data has more than five years.
- The ES classification system itself can be a source of uncertainty because of the ambiguity present in some classes, as previously referred. Some ES are difficult to assign to specific spatial units. For example, *‘aesthetic’* class and *‘other cultural outputs’* group within cultural services are appreciated in a very subjective manner and related to the landscape compositions^47^ and other social factors (e.g., cultural).
- Assumptions made in the course of the mapping, for example regarding the ecological status of the biotope and its ability to provide a certain ES.

## Conclusions

The proposed framework proved to be suitable for addressing ES in complex coastal regions. On the one hand, it uses clear and objective criteria for delineating the geographic area, respecting the connectivity of natural systems but also the complexity of the governance framework, usual in these systems. Therefore, the analysis at a regional scale and the integration of several ecosystem typologies is seen as crucial for such socio-ecological systems. The use of the internationally accepted CICES classification, although adapted to the case study reality and scale, is seen as an advantage, allowing comparisons with other studies. On the other hand, the fact that the mapping approach is based on existing and available data, considers a wide range of ES and abiotic outputs, and uses mainstream software, means that this approach should be somehow easily replicable by technicians, and planners without investing large amounts of human and economic resources.

Exploring the application of CICES and an ES mapping approach based on qualitative indicators to Ria de Aveiro coastal region also allowed the identification of a number of pertinent issues. For instance, because the classes within regulation and maintenance services are relatively specific and require scientific-specific knowledge about biological and physic-chemical processes, CICES application by decision-makers, technicians, and planners can be demanding. Regarding the mapping approach, inspiration, and sense of place services within the ES class *‘aesthetic’* provided by the Ria de Aveiro coastal region, which is deeply present in the region^30^, still remains a challenge. Additionally, the mapping approach assumes that every part of a given ecosystem is of equal value with regard to its capacity to provide ES, without taking into consideration the ecosystems’ health, or the fact that certain habitats might have comparatively higher or lower potential to provide a certain service. The integration and analysis of additional information (e.g., ecosystem quality status data, the design of rules that could help grade the importance of different habitats capacity in providing ES) in the mapping process opens further opportunities.

## Additional Information

**How to cite this article**: Sousa, L. P. *et al.* Ecosystem services provided by a complex coastal region: challenges of classification and mapping. *Sci. Rep.* **6**, 22782; doi: 10.1038/srep22782 (2016).

## Supplementary Material

Supplementary Information

## Figures and Tables

**Figure 1:**
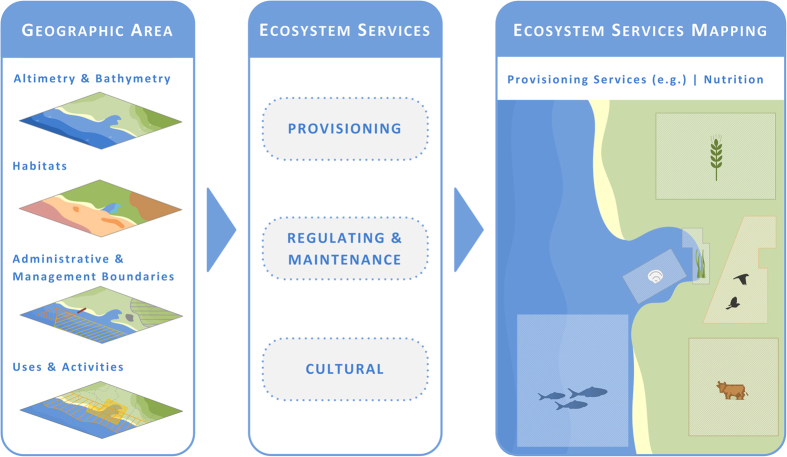
Overview of the conceptual approach: From the boundaries definition to the ES classification and mapping. Drawings were generated with Microsoft Visio Professional 2016.

**Table 1:** ES definitions (adapted from Vandewalle *et al.* 49 Braat and de Groot^19^ Häyhä and Franzese^20^).

Source	ES definition	Ecosystems	Economic
Daily^50^	The conditions and processes through which natural ecosystems, and the species that make them up, sustain and fulfil human life	•	
Costanza *et al.*^51^	The benefits human populations derive, directly or indirectly, from ecosystem functions		•
MA^23^	The benefits people obtain from ecosystems	•	•
Boyd and Banzhaf^21^	Components of nature, directly enjoyed, consumed, or used to yield human well-being		•
Fisher *et al.*^22^	The aspects of ecosystems utilized (actively or passively) to produce human well-being		•
TEEB^24^	The direct and indirect contributions of ecosystems to human well-being	•	•
Haines‐Young and Potschin^11^	The contributions that ecosystems make to human well-being	•	•

**Table 2:** Summary of the ES provided by the case study area (CW denotes coastal waters; TW denotes transitional waters; FW denotes freshwaters; TE denotes terrestrial ecosystems (including agro-ecosystems); • denotes presence of the ES; *stands for adaptations of CICES V4.3 during the mapping process).

Section	Division	Group	Class	CW	TW	FW	TE
PROVISIONING	Nutrition	Biomass	Cultivated crops				•
			Reared animals and their outputs				•
			Wild plants, algae and their outputs		•	•	
			Wild animals and their outputs	•	•	•	
			Plants and algae from in-situ aquaculture		•		
			Animals from in-situ aquaculture		•		
		Water	Surface water for drinking				
			Ground water for drinking				
	Materials	Biomass	Fibres and other materials from plants, algae and animals for direct use or processing		•	•	•
			Materials from plants, algae and animals for agricultural use		•	•	•
			Genetic materials from all biota				•
		Water	Surface water for non-drinking purposes		•	•	
			Ground water for non-drinking purposes			•	
	Energy	Biomass-based energy sources	Plant-based resources				
			Animal-based resources				
		Mechanical energy	Animal-based energy				•
REGULATION & MAINTENANCE	Mediation of waste, toxics and other nuisances	Mediation by biota	Bio-remediation by micro-organisms, algae, plants, and animals	•	•	•	•
			Filtration/sequestration/storage/accumulation by micro-organisms, algae, plants, and animals*	•	•	•	•
		Mediation by ecosystems	Filtration/sequestration/storage/accumulation by ecosystems	•	•	•	•
			Dilution by atmosphere, freshwater and marine ecosystems	•	•	•	
			Mediation of smell/noise/visual impacts				•
	Mediation of flows	Mass flows	Mass stabilisation and control of erosion rates		•	•	•
			Buffering and attenuation of mass flows	•	•	•	
		Liquid flows	Hydrological cycle and water flow maintenance		•	•	•
			Flood protection		•	•	•
		Gaseous / air flows	Storm protection				
			Ventilation and transpiration				•
	Maintenance of physical, chemical, biological conditions	Lifecycle maintenance, habitat and gene pool protection	Pollination and seed dispersal			•	•
			Maintaining nursery populations and habitats	•	•	•	•
		Pest and disease control	Pest control*	•	•	•	•
			Disease control				
		Soil formation and composition	Weathering processes				•
			Decomposition and fixing processes	•	•	•	•
		Water conditions	Chemical conditions of freshwaters*	•	•	•	
			Chemical conditions of salt waters*	•	•	•	
		Atmospheric composition and climate regulation	Global climate regulation by reduction of greenhouse gas concentrations	•	•	•	•
			Micro and regional climate regulation				•
CULTURAL	Physical and intellectual interactions with biota, ecosystems, and land- /seascapes [environmental settings]	Physical and experiential interactions	Experiential use of plants, animals and land-/seascapes in different environmental settings		•	•	•
			Physical use of land-/seascapes in different environmental settings	•	•	•	
		Intellectual and representational interactions	Scientific	•	•	•	•
			Educational		•	•	•
			Heritage, cultural		•	•	•
			Entertainment		•	•	•
			Aesthetic	•	•	•	•
	Spiritual, symbolic and other interactions with biota, ecosystems, and land- /seascapes [environmental settings]	Spiritual and/or emblematic	Symbolic				
			Sacred and/or religious				
		Other cultural outputs	Existence*	•	•	•	•
			Bequest*	•	•	•	•

**Table 3:** Summary of the indicators used to map the ES and abiotic outputs provided by the case study (NA denotes not applicable).

Section	Division	Group	Class	Indicator
PROVISIONING	Nutrition	Biomass	Cultivated crops	Presence of annual crops, rice fields and “bocage”
			Reared animals and their outputs	Presence of pastures and “bocage”
			Wild plants, algae and their outputs	Presence of authorized collecting areas
			Wild animals and their outputs	Presence of fishing zones, shellfish collecting areas, hunting areas
			Plants and algae from in-situ aquaculture	Presence of active units
			Animals from in-situ aquaculture	Presence of active units
	Materials	Biomass	Fibres and other materials from plants, algae and animals for direct use or processing	Presence of reed marshes along Ria de Aveiro, mudflats, and forested habitats
			Materials from plants, algae and animals for agricultural use	Presence of *Zostera noltei* bed habitat subgroup and rush marsh
			Genetic materials from all biota	Presence of “bocage”
		Water	Surface water for non-drinking purposes	Presence of rivers, ditches, freshwater lakes, aquaculture, active salt pans, transitional waters, and water scooper operation areas
			Ground water for non-drinking purposes	Presence of groundwater abstraction points
	Energy	Mechanical energy	Animal-based energy	Presence of pastures and “bocage”
REGULATION & MAINTENANCE	Mediation of waste, toxics and other nuisances	Mediation by biota	Bio-remediation by micro-organisms, algae, plants, and animals	All the considered habitats (e.g. intertidal flats, soils, aquatic and terrestrial vegetated areas)
		Mediation by ecosystems	Filtration/sequestration/storage/accumulation by biota and ecosystems	Presence of salt marshes, reed marshes, intertidal flats (including *Zostera noltei* beds), coastal waters, riparian and alluvial forests
			Dilution by atmosphere, freshwater and marine ecosystems	Presence of coastal waters, transitional waters and freshwaters
			Mediation of smell/noise/visual impacts	Presence of “bocage”
	Mediation of flows	Mass flows	Mass stabilisation and control of erosion rates	Presence of coastal dunes, salt marshes, reed marshes, *Zostera noltei* beds, forests (including alluvial and riparian forest), natural grassland, and shrubland
			Buffering and attenuation of mass flows	Presence of salt marshes, reed marshes, *Zostera noltei* beds, coastal waters, transitional waters, and freshwaters
		Liquid flows	Hydrological cycle and water flow maintenance	Presence of riparian forest, salt marshes and other areas with high evapotranspiration
			Flood protection	Presence of coastal dunes, salt marshes, reed marshes, riparian forest, and “bocage”
		Gaseous/air flows	Ventilation and transpiration	Presence of “bocage”
	Maintenance of physical, chemical, biological conditions	Lifecycle maintenance, habitat and gene pool protection	Pollination and seed dispersal	Presence of forests (including alluvial and riparian forest), and “bocage” along low lands of Vouga river
			Maintaining nursery populations and habitats	Presence of rivers, freshwater lakes, transitional waters, salt pans, salt marshes, reed marshes, intertidal flats (including *Zostera noltei* beds), coastal waters, “bocage”, fixed dunes with herbaceous vegetation, and dunes with *Salix*, and forests (including alluvial and riparian forest)
		Soil formation and composition	Weathering processes	Presence of fluvisols combined with forests and floodplain areas
			Decomposition and fixing processes	All the considered habitats (e.g. intertidal flats, soils, aquatic and terrestrial vegetated areas)
		Atmospheric composition and climate regulation	Global climate regulation by reduction of greenhouse gas concentrations	Presence of coastal waters, forests (including alluvial and riparian forest), forested dunes, salt marshes, reed marshes, and *Zostera noltei* beds
			Micro and regional climate regulation	Presence of “bocage”
CULTURAL	Physical and intellectual interactions with biota, ecosystems, and land- /seascapes [environmental settings]	Physical and experiential interactions	Experiential use of plants, animals and land-/seascapes in different environmental settings	Designated places for birdwatching and land-/seascape appreciation
			Physical use of land-/seascapes in different environmental settings	Area of activity (e.g. sailing, canoeing, surfing)
		Intellectual and representative interactions	Scientific	Territory subject of scientific research
			Educational	Location of eco-museums, and environmental interpretative centres
			Heritage, cultural	Designated subaquatic archaeological sites, location of buildings with traditional architecture, and location of traditional activities
			Entertainment	Location of the festivals and fairs
			Aesthetic	Location of permanent artistic exhibitions
ABIOTIC OUTPUTS	Nutritional abiotic substances	Mineral	NA	Presence of active salt pans
	Abiotic materials	Non-metallic	NA	Designated areas for sand and gravel exploitation
	Maintenance of physical, chemical, abiotic conditions	By natural chemical and physical processes	NA	Presence of transitional waters, rivers, and freshwater lakes
