# Supplementary material for: Ecosystem services provided by a complex coastal region: challenges of classification and mapping
Source: Sci Rep. 2016 Mar 11;6:22782. doi: 10.1038/srep22782 (PMC4786800; doi:10.1038/srep22782)
Supplement: Supplementary Information [file srep22782-s1.pdf]

Supplementary Information for

Ecosystem services provided by a complex coastal region: challenges of classification and mapping

Lisa P. Sousa[1*], Ana I. Sousa [2], Fátima L. Alves[1], Ana I. Lillebø [2]

[1] Department of Environment and Planning & CESAM - Centre for Environmental and Marine Studies, University of Aveiro, Campus Universitário de Santiago, 3810-193 Aveiro, Portugal

[2] Department of Biology & CESAM - Centre for Environmental and Marine Studies, University of Aveiro, Campus Universitário de Santiago, 3810-193 Aveiro, Portugal

* Corresponding author: lisa@ua.pt

Supplementary Table S1. Summary of the indicators used for mapping the ES provided by the case study (LU/LC denotes land use/land cover; NA denotes not applicable).

ES Class (CICES)	Ria de Aveiro ES description	Indicator	Typology of data	Data source
Provisioning				
Cultivated crops	Annual crops (e.g., soy, beans, corn, wheat, rice), fruits, vegetables, and forage	Presence of annual crops, rice fields and “bocage”	LU/LC; Habitat units	COS 2007[1]; AMBIECO/PLRA, 2011[2]
Reared animals and their outputs	Meat (e.g., “marinhoa” cattle) and dairy products (milk, cheese, yogurt)	Presence of pastures and “bocage”	LU/LC; Habitat units	COS 2007[1]; AMBIECO/PLRA, 2011[2]
Wild plants, algae and their outputs	Wild glasswort *Salicornia* sp.	Presence of authorized collecting area	Administrative and legal processes	
Wild animals and their outputs	Fisheries: freshwater (e.g., lamprey, allis shad, twaite shad); brackishwater (e.g., lamprey, european eel, allis shad, cuttlefish); seawater (e.g., atlantic horse mackerel, sardine); shellfish (e.g., spinous spider crab, clams, cockle, mussels)	Presence of fishing zones, shellfish collecting areas	Habitat units; Administrative and legal processes	AMBIECO/PLRA, 2011[2]; APAveiro, 2012[3]
	Game: wild ducks, quails and doves	Presence of hunting areas	(No spatial data available)	-
Plants and algae from in-situ aquaculture	In-situ macroalgae farming (*Gracilaria verrucosa, Chondrus crispus, Ulva lactuca, Porphyra* spp.*, Codium tomentosum*)	Presence of active units	Location of the activity	AlgaPlus, 2014[4]
Animals from in-situ aquaculture	In-situ aquaculture farms of marine fish (e.g. gilthead seabream - *Sparus aurata*, seabass - *Dicentrarchus labrax,* and turbot - *Psetta maxima*) and shellfish (Japanese oyster - *Crassostrea gigas*, clams - *Ruditapes decussates*)	Presence of active units	Habitat units	AMBIECO/PLRA, 2011[2]; Aerial photograph (ESRI basemap)
Surface water for drinking	NA	NA	NA	NA
Ground water for drinking	NA	NA	NA	NA
Fibres and other materials from plants, algae and animals for direct use or processing	Reeds are harvested and used for traditional products/handcraft (e.g. mats/dunnage)	Presence of reed marshes along Ria de Aveiro	Habitat units	AMBIECO/PLRA, 2011[2]
	Solitary tube worm (*Diopatra neapolitana*, “casulo”), ragworm (*Hediste diversicolor*) and catworm (*Nephthys hombergii*) are collected to be use as bait for fishing	Presence of mudflats	Habitat units	AMBIECO/PLRA, 2011[2]
	Wood and timber for industrial use (e.g. cellulose for paper)	Presence of forested habitats	LU/LC; Habitat units	COS 2007[1]; AMBIECO/PLRA,

ES Class (CICES)	Ria de Aveiro ES description	Indicator	Typology of data	Data source
				2011[2]
Materials from plants, algae and animals for agricultural use	Seagrasses and macroalgae ("moliço") are harvested to be used as fertilizers in agriculture	Presence of *Zostera noltei* bed	Habitat units	AMBIECO/PLRA, 2011[2]
	Rush marshes (*Juncus maritimus*) are harvested and used as cattle bedding and afterwards as fertilizer, as raw materials for mats, and for protecting salt mounds from wind and rain	Presence of rush marsh	Habitat units	AMBIECO/PLRA, 2011[2]
Genetic materials from all biota	"Marinhoa" cattle (registered as Protected Designations of Origin - PDO)	Presence of "bocage"	Habitat units	AMBIECO/PLRA, 2011[2]
Surface water for non-drinking purposes	Surface water is abstracted from the coastal lagoon, Pateira de Fermentelos lake and freshwater systems for forest-fire control, crops irrigation and livestock consumption, aquaculture and salt production, and for industrial use	Presence of rivers, ditches, freshwater lakes, aquaculture, active salt pans, transitional waters, and water scooper operation areas	Habitat units; legal instruments	AMBIECO/PLRA, 2011[2]; INAG, 2011[5]; ADAPT-MED, 2013[6]; APAveiro, 2012[3]; CM Águeda, 2014[7]
Ground water for non-drinking purposes	Groundwater abstraction for public supply from the "Cretácico de Aveiro" and "Quaternário de Aveiro"	Presence of groundwater abstraction points	Legal instruments	RCM 95/2007, 23 July
Plant-based resources	NA	NA	NA	NA
Animal-based resources	NA	NA	NA	NA
Animal-based energy	Use of "Marinhoa" cattle in the agriculture	Presence of pastures and "bocage"	LU/LC; Habitat units	COS 2007[1]; AMBIECO/PLRA, 2011[2]
Regulation and Maintenance				
Bio-remediation by micro-organisms, algae, plants, and animals	Biological filtration by micro-organisms, algae, plants, and animals (e.g. oysters, clams and mussels)	All the considered habitats (e.g. intertidal flats, soils, aquatic and terrestrial vegetated areas)	Habitat units	AMBIECO/PLRA, 2011[2]
Filtration/ sequestration/ storage/ accumulation by biota and ecosystems	Bio-physicochemical filtration/ sequestration/ storage/ accumulation of pollutants by macrophytes; adsorption and binding of metals and organic compounds in ecosystems, as a result of combination of biotic and abiotic factors	Presence of salt marshes, reed marshes, intertidal flats (including *Zostera noltei* beds), and coastal waters	Habitat units	AMBIECO/PLRA, 2011[2]
	Riparian areas maintain/protect water quality by capturing and filtrating water through their soils before it gets to streams	Presence of riparian and alluvial forests	LU/LC; Habitat units	COS 2007[1]; AMBIECO/PLRA, 2011[2]

ES Class (CICES)	Ria de Aveiro ES description	Indicator	Typology of data	Data source
Dilution by atmosphere, freshwater and marine ecosystems	Bio-physicochemical dilution of gases, fluids and solid waste, wastewater in sea, rivers, lakes and the lagoon	Presence of coastal waters, transitional waters and freshwaters	Habitat units	AMBIECO/PLRA, 2011[2]; INAG, 2011[5]
Mediation of smell/noise/visual impacts	"Bocage", as green infrastructure, reduces the visual impact and the smell from a pulp mill industry	Presence of "bocage"	Habitat units	AMBIECO/PLRA, 2011[2]
Mass stabilisation and control of erosion rates	Dunes, saltmarshes and seagrass beds help to maintain the lagoon integrity. Dune vegetation is crucial to its formation and coastline stabilisation	Presence of coastal dunes (also with *Acacia* sp.*)*, salt marshes (including rush marshes), reed marshes, *Zostera noltei* beds	LU/LC; Habitat units	COS 2007[1]; AMBIECO/PLRA, 2011[2]
	Riparian areas are essential for bank stabilisation and erosion protection. "Bocage" contributes to erosion reduction	Presence of riparian and alluvial forests, "bocage"	LU/LC; Habitat units	COS 2007[1]; AMBIECO/PLRA, 2011[2]
	Overall vegetation cover helps to stabilise terrestrial ecosystems	Presence of forests, natural grassland, and shrubland	LU/LC; Habitat units	COS 2007[1]; AMBIECO/PLRA, 2011[2]
Buffering and attenuation of mass flows	Seagrass meadows and salt marshes reduce sediment re-suspension and turbidity in the water column, contributing to increase the light availability in the water column.	Presence of salt marshes, reed marshes, and *Zostera noltei* beds	Habitat units	AMBIECO/PLRA, 2011[2]
	Transport and storage of sediment by rivers, lakes, coastal lagoons and the ocean	Presence of coastal water, transitional water, and freshwater	Habitat units	AMBIECO/PLRA, 2011[2]; INAG, 2011[5]
Hydrological cycle and water flow maintenance	Riparian areas have the capacity to slow/reduce the water flow and store it for future use	Presence of riparian forest	LU/LC; Habitat units	COS 2007[1]; AMBIECO/PLRA, 2011[2]
	Salt marshes have a significant influence on the hydrological cycle	Presence of salt marshes habitat units	LU/LC; Habitat units	AMBIECO/PLRA, 2011[2]
	Present in the areas where evapotranspiration is higher, which in this case coincide with "bocage" and forest (excluding transitional grass habitats)	Areas with high evapotranspiration	Evapotranspiration	LAGOONS, 2013[8]
Flood protection	Appropriate land coverage provide resilience to extreme weather events and act as physical buffering of climate change	Presence of coastal dunes, salt marshes, reed marshes, riparian forest, and "bocage"	LU/LC; Habitat units	COS 2007[1]; AMBIECO/PLRA, 2011[2]

ES Class (CICES)	Ria de Aveiro ES description	Indicator	Typology of data	Data source
Storm protection	NA	NA	NA	NA
Ventilation and transpiration	“Bocage” enables air ventilation	Presence of “bocage”	Habitat units	AMBIECO/PLRA, 2011[2]
Pollination and seed dispersal	Vegetation features supporting pollination	Presence of forests (including alluvial and riparian forest), and “bocage” along low lands of Vouga river	LU/LC; Habitat units	COS 2007[1]; AMBIECO/PLRA, 2011[2]
Maintaining nursery populations and habitats	Vouga, Águeda and Levira rivers are relevant spawning areas for anadromous migratory species and *Lampetra planeri*	Presence of rivers and freshwater lakes	Habitat units	AMBIECO/PLRA, 2011[2]; INAG, 2011[5]; RCM no. 1125-A/2008, 21 July; MESHAtlantic, 2014[9]
	Ria de Aveiro is a nursery habitat for fisheries and invertebrates	Presence of transitional waters, salt pans, salt marshes, intertidal flats (including *Zostera noltei* beds)		
	Coastal waters is an important habitat for fisheries	Presence of coastal waters		
	“Bocage” and salt pans are important area for birds feeding and breeding	Presence of “bocage”	Habitat units	AMBIECO/PLRA, 2011[2]
	Fixed coastal dunes with herbaceous vegetation provide shelter for biodiversity	Presence of fixed dunes with herbaceous vegetation, and dunes with Salix	Habitat units	AMBIECO/PLRA, 2011[2]
	The study area provides other important habitats such as reeds, riparian, alluvial, and other forests.	Presence of forests (including alluvial and riparian forest), and reed marshes	LU/LC; Habitat units	COS 2007[1]; COS 2007[1]; AMBIECO/PLRA, 2011[2]
Pest control	Note: see discussion section of the manuscript	-	-	-
Disease control	NA	NA	NA	NA
Weathering processes	Fluvisols are the type of soils with higher level/content of organic matter. Floodplains constitute important sinks of river nutrients and sediments (transported during flood events), which contribute to the maintenance of soil fertility and nutrient storage	Presence of fluvisols combined with forests and floodplain areas	LU/LC; Soil map	COS 2007[1]; Atlas do Ambiente, 1982[10]
Decomposition and fixing processes	Nitrogen cycling (nitrogen fixing, denitrification, decomposition) in intertidal mudflats, seagrass meadows and salt marshes.	All the considered habitats (e.g. intertidal flats, soils, aquatic and	LU/LC; Habitat units	COS 2007[1]; AMBIECO/PLRA, 2011[2]

ES Class (CICES)	Ria de Aveiro ES description	Indicator	Typology of data	Data source
	Terrestrial ecosystems contribute to the maintenance of bio-geochemical conditions of soils by decomposition/mineralisation of dead organic material, nitrification and denitrification	terrestrial vegetated areas)		
Chemical conditions of freshwaters	Note: see discussion section of the manuscript	-	-	-
Chemical conditions of salt waters	Note: see discussion section of the manuscript	-	-	-
Global climate regulation by reduction of greenhouse gas concentrations	Fixation of atmospheric carbon by oceanic algae and its eventual deposition in deep water represents an important part of the global carbon cycle and thus influences climate trends	Presence of coastal water	Habitat units	INAG, 2011[5]
	Global climate regulation by greenhouse gas/carbon sequestration by terrestrial ecosystems, water columns and sediments and their biota	Presence of forests (including alluvial and riparian forest), forested dunes, salt marshes, reed marshes and *Zostera noltei* beds	LU/LC; Habitat units	COS 2007[1]; AMBIECO/PLRA, 2011[2]
Micro and regional climate regulation	Green infrastructures contribute to the control of atmospheric conditions (e.g., temperature, humidity and wind)	Presence of "bocage"	Habitat units	AMBIECO/PLRA, 2011[2]; INAG, 2011[5]
Cultural				
Experiential use of plants, animals and land-/seascapes in different environmental settings	Birdwatching and land-/seascape appreciation (e.g. natural and semi-natural beaches, salt pans, quays, public gardens along rivers and lakes, city channels, Ria's islands, São Jacinto dunes Nature Reserve and Baixo Vouga Lagunar)	Designated places for birdwatching and land-/seascape appreciation	Viewpoints, birdwatching points, protected areas	AMBIECO/PLRA, 2011[2]; Turismo Centro de Portugal, 2015[11]; PLRA, 2010[12]; ICNF, 2014[13]; POC OMG, 2015[14]; CM Ílhavo, 2015[15]; CM Albergaria-a-Velha[16]
Physical use of land-/seascapes in different environmental settings	Sailing, canoeing, rowing, swimming, surfing, windsurfing, kitesurfing, cycling, walking, leisure fishing and hunting	Area of activity	Leisure and sports data	POEM, 2012[17]; POC OMG, 2015[14]; PLRA, 2010[12]; Turismo Centro de Portugal, 2015[11]; CCDRC, 2015[18]; CM Águeda, 2015[19]; CM Ílhavo, 2015[15]; CM Estarreja, 2015[20]; CM Murtosa, 2015[21]; CM Aveiro, 2015[22]; BIORIA, 2014[23]; APAveiro, 2014[24]

ES Class (CICES)	Ria de Aveiro ES description	Indicator	Typology of data	Data source
Scientific	The entire study area in subject matter of research	Territory subject of scientific research	Study areas	FCT, 2015[25]; Research Centres; WOS, 2015[26]
Educational	Natural and cultural heritage of the study area are subject matter of education	Location of eco-museums, and environmental interpretative centres	Museological infrastructures; environmental interpretative centres	CCDRC, 2015[18]; Turismo Centro de Portugal, 2015[11]
Heritage, cultural	Subaquatic archaeological sites in the lagoon (e.g. shipwrecks, ship hull, and other isolated findings)	Designated subaquatic archaeological sites	Legal instruments	DGPC, 2014[27]
	Traditional architecture (e.g. “palheiros”, “Gafanhoa”), traditional boats (e.g. “moliceiro”, “bateira”, “mercantel”) and traditional activities (e.g. salt production, “arte Xávega”)	Location of buildings with traditional architecture, and location of traditional activities	Vernacular heritage, and Intangible cultural activities	Turismo Centro de Portugal, 2015[11]
Entertainment	Ex-situ experiences through festivals related with the activities and products of the study area (e.g. gastronomic fairs, Vagueira surf festival, Ria de Aveiro Weekend, ObservaRia, “moliceiro” feast, NªSª Navegantes fair)	Location of the festivals and fairs	Intangible cultural heritage	CM Águeda, 2015[19]; CM Ílhavo, 2015[15]; CM Estarreja, 2015[20]; CM Murtosa, 2015[21]; CM Aveiro, 2015[22]; CM Vagos[28]; CM Ovar[29]; BIORIA, 2014[23]
Aesthetic	Artistic representations of nature and related activities (e.g. public monuments, statues, tile murals, ceramic tiles, painted shells)	Location of permanent artistic exhibitions	Cultural heritage	CM Ílhavo, 2015[15]; CM Murtosa, 2015[21]; CM Aveiro, 2015[22]
	Inspiration for some painters and writers, interested in the history and heritage of the lagoon and its users	-	-	-
	Sense of place	-	-	-
Symbolic	NA	NA	NA	NA
Sacred and/or religious	NA	NA	NA	NA

Supplementary Table S2. Summary of the indicators for mapping the abiotic outputs provided by the case study (NA denotes not applicable).

ES Division (CICES)	ES Group (CICES)	Ria de Aveiro ES description	Indicator	Typology of data	Data source
Abiotic provisioning					
Nutritional abiotic substances	Mineral	Salt production	Presence of active salt pans	Habitat units	AMBIECO/ PLRA, 2011[2]
	Non-mineral	NA	NA	NA	NA
Abiotic materials	Metallic	NA	NA	NA	NA
	Non-metallic	Occurrence of exploitable sand and gravel	Designated areas for sand and gravel exploitation	Legal instruments	POEM, 2012[17]
Energy	Renewable abiotic energy sources	NA	NA	NA	NA
	Non-renewable energy sources	NA	NA	NA	NA
Regulation & Maintenance by natural physical structures and processes					
Mediation of waste, toxics and other nuisances	By natural chemical and physical processes	NA	NA	NA	NA
Mediation of flows by natural abiotic structures	By solid (mass), liquid and gaseous (air)flows	NA	NA	NA	NA
Maintenance of physical, chemical, abiotic conditions	By natural chemical and physical processes	Blue infrastructures contribute to weather regulation	Presence of transitional waters, rivers, and freshwater lakes	Habitat units	AMBIECO/ PLRA, 2011[2]
Cultural settings dependent on abiotic structures					
Physical and intellectual interactions with land-/seascapes [physical settings]	By physical and experiential interactions or intellectual and representational interactions	NA	NA	NA	NA
Spiritual, symbolic and other interactions with land-/seascapes [physical settings]	By type	NA	NA	NA	NA

Supplementary Table S3. Habitat reclassification (* indicates the habitat confirmed by field work by AMBIECO/PLRA[2])

Habitat or land cover group	Habitat or land cover subgroup	Habitat code
Freshwater	Permanent eutrophic water bodies	3150
	Freshwater lakes	
	River	
Coastal lagoons	Transitional waters	1150
Low salt marsh	*Spartina* swards	1320*
	Halophytic vegetation	1310pt1*
Medium & hight salt marsh	Atlantic salt meadows	1330+1320*
Intertidal flats	Sandflats	1140pt1*
	Mudflats	1140pt1*
	Zostera noltei beds	1140pt2*
Salt pans	Active salt pans	
	Destroyed salt pans	
	Flooded salt pans	
Rush marsh	Rush marsh	1410
Reed marsh	Reed marsh	
Beaches and sands	Maritime beaches and sands	1110
	Inland beaches and sands	
Coastal dunes	Dunes with *Salix*	2170*
	Forested dune	2180, 2270
	Fixed dunes with herbaceous vegetation ('grey dunes')	2130*
	Shifting dunes with *Ammophila arenaria* ('white dunes')	2120*
Coastal waters	Infralittoral fine sand	A5.23
	Circalittoral fine sand	A5.25
	Infralittoral mixed sediments	A5.43
	Circalittoral mixed sediments	A5.44
	Infralittoral muddy sand	A5.24
Acacia	*Acacia*	
Forests	Oak tree forest	9230
	Poplar forest	
	Other forests	
	Broad-leaved forest	

Habitat or land cover group	Habitat or land cover subgroup	Habitat code
	Pine, Eucalyptus, Acacia	
Alluvial forests	Alder riparian forest	91E0pt1*
	Alder swamp forest	91E0pt3*
Riparian forest	Riparian mixed forest	91F0*
	Other riparian areas	
Natural grassland	Shrubland	
	Natural grasslands	
Aquaculture	Aquaculture	NA
Agricultural areas	Rice fields	NA
	“Bocage”	NA
	Annual crops	NA
Artificial surfaces	Built-up areas	NA
	Green urban areas	NA

References

1. Instituto Geográfico Português. *Carta de Uso e Ocupação do Solo de Portugal Continental para 2007 (COS 2007)*. (2010).

2. AMBIECO/PLRA. *Estudo da Caracterização da Qualidade Ecológica da Ria de Aveiro*. *Ria de Aveiro POLIS LITORAL, AMBIECO* (2011).

3. Capitania do Porto de Aveiro. *Edital n.º 1/2012*. (2012).

4. AlgaPlus. (2014). at <http://www.algaplus.pt/>

5. Instituto da Água. *Massas de água - DQA*. (2011).

6. ADAPT-MED. *D2.1b - Baixo Vouga Lagunar Knowledge Database*. (2013).

7. Câmara Municipal de Águeda. *Pontos de água*. (2014). at <http://softwarelivre.cm-agueda.pt/drupal/?q=node/4>

8. LAGOONS. *D5.1 - Results of Climate impact assessment. Application for four lagoon catchments*. (2013).

9. MESHAtlantic. *Predicted broad-scale EUNIS habitats - Atlantic area*. Published on 10 December 2013, updated on 11th February 2014. (2014). at <http://www.emodnet-seabedhabitats.eu/download>

10. Agência Portuguesa do Ambiente. Carta de Solos. *Atlas Digital do Ambiente* (1982). at <http://sniamb.apambiente.pt/Home/Default.htm>

11. Turismo Centro de Portugal. *SIG Ria de Aveiro*. (2013). at <http://sig.riadeaveiro.pt/web/>

12. POLIS LITORAL Ria de Aveiro. *Estudo de Caracterização para o Reordenamento e Valorização dos Núcleos Piscatórios Lagunares, no âmbito do Polis Litoral Ria de Aveiro – Relatório Final*. (2010).

13. Instituto da Conservação da Natureza e das Florestas. *Áreas Protegidas*. (2014). at <http://www.icnf.pt/portal/naturaclas/cart/ap-rn-ramsar-pt>

14. Agência Portuguesa do Ambiente. *Programa de Orla Costeira Ovar – Marinha Grande (POC-OMG)*. (2015).

15. Câmara Municipal de Ílhavo. at <http://www.cm-ilhavo.pt/>

16. Câmara Municipal de Albergaria-a-Velha. at <http://www.cm-albergaria.pt/>

17. Direção-Geral de Política do Mar. *Proposta de Plano de Ordenamento do Espaço Marítimo*. (2012).

18. Comissão de Coordenação e Desenvolvimento Regional do Centro. *Roteiro dos museus e espaços museológicos na Região Centro*. at <http://roteiromuseus.ccdrc.pt/>

19. Câmara Municipal de Águeda. at <https://www.cm-agueda.pt/>

20. Câmara Municipal de Estarreja. at <http://www.cm-estarreja.pt/>

21. Câmara Municipal de Murtosa. at <http://www.cm-murtosa.pt/>

22. Câmara Municipal de Aveiro. at <http://www.cm-aveiro.pt/www/>

23. BIORIA. *Percursos*. at <http://www.bioria.com/>

24. Administração do Porto de Aveiro S.A. *Normas de Segurança Marítima e Portuária do Porto de Aveiro*. (2014).

25. Fundação para a Ciência e a Tecnologia. *Projectos de I&D*. at <http://www.fct.pt/apoios/projectos/consulta/projectos>

26. WOS. *Web of Science*. at <www.webofknowledge.com>

27. Direção-Geral do Património Cultural. *Arqueologia Náutica e Subaquática*. at <http://www.patrimoniocultural.pt/pt/>

28. Câmara Municipal de Vagos. at <http://www.cm-vagos.pt/PageGen.aspx>

29. Câmara Municipal de Ovar. at <https://www.cm-ovar.pt>
